# The Efficacy of First-Line Pembrolizumab Monotherapy in Patients with Metastatic NSCLC Aged ≥70 Years with High PD-L1 (TPS ≥ 50%) Expression: A Multicenter Real-World Study

**DOI:** 10.3390/cancers17132190

**Published:** 2025-06-28

**Authors:** Filip Marković, Maximilian Hochmair, Nino Müser, Hannah Fabikan, Vania Mikaela Rodriguez, Urska Janzic, Mihailo Stjepanović, Milica Kontić

**Affiliations:** 1 Clinic for Pulmonology, University Clinical Center of Serbia, 11000 Belgrade, Serbia; mihailo.stjepanovic@kcs.ac.rs; 2 Department of Respiratory and Critical Care Medicine, Clinic Floridsdorf, Vienna Healthcare Group, 1130 Vienna, Austria; maximilian.hochmair@gesundheitsverbund.at; 3 Karl Landsteiner Institute of Lung Research and Pulmonary Oncology, Clinic Floridsdorf, 1210 Vienna, Austria; hannah.fabikan@extern.gesundheitsverbund.at (H.F.); vaniamikaela.rodriguez@extern.gesundheitsverbund.at (V.M.R.); 4 Karl Landsteiner Institute of Lung Research and Pulmonary Oncology, 2nd Department of Internal Medicine with Pulmonology, Klinik Ottakring, Vienna Healthcare Group, 1210 Vienna, Austria; nino.mueser@gesundheitsverbund.at; 5 Medical Oncology Unit, University Clinic Golnik, 4204 Golnik, Slovenia; urska.janzic@klinika-golnik.si; 6 Medical Faculty, University of Ljubljana, 1000 Ljubljana, Slovenia; 7 School of Medicine, University of Belgrade, 11000 Belgrade, Serbia

**Keywords:** non-small-cell lung cancer, pembrolizumab, elderly, PD-L1, smoking, real-world data

## Abstract

**Introduction:** Elderly patients with metastatic non-small-cell lung cancer (NSCLC) are underrepresented in clinical trials evaluating immune checkpoint inhibitors (ICIs). This study assesses the efficacy of first-line pembrolizumab monotherapy in patients with metastatic NSCLC aged ≥70 years with a programmed death-ligand 1 (PD-L1) tumor proportion score (TPS) ≥ 50%. **Materials and Methods:** We retrospectively analyzed 381 patients with metastatic NSCLC without oncogenic driver mutations treated with pembrolizumab monotherapy between 2017 and 2023 at three academic centers in Central and Southeastern Europe. Clinical outcomes, including median time on treatment (mToT) and overall survival (mOS), were compared between patients aged ≥70 and <70 years. **Results:** Of 381 patients, 149 (39.1%) were aged ≥70. No significant differences in mToT (12.7 vs. 14.3 months; *p* = 0.125) or mOS (18.2 vs. 27.4 months; *p* = 0.124) were observed between older and younger groups. Good ECOG PS (0–1) was independently associated with longer mToT and mOS in older patients. Additionally, a history of smoking was linked to improved outcomes compared to never-smokers, suggesting potential immunogenic effects. Older patients were significantly less likely to receive second-line therapy after progression (*p* = 0.04). **Conclusions:** First-line pembrolizumab monotherapy is effective across all age groups and provides similar treatment efficacy in patients with metastatic NSCLC and a PD-L1 TPS ≥ 50%. Patients older than 70 with a smoking history and ECOG PS 0–1 derive the most benefit from this treatment modality. The adaptation of this treatment strategy for elderly patients is especially important, since only a minority of them are capable of receiving second-line therapy.

## Simple Summary

This real-world study assessed the effectiveness of first-line pembrolizumab monotherapy in patients with metastatic non-oncogene-addicted NSCLC and PD-L1 TPS ≥ 50%, comparing those aged ≥70 years to younger patients. Results showed no significant differences in outcomes—objective response rate, time on treatment (ToT), and overall survival (OS)—between the two age groups. In older patients, a history of smoking and good performance status (ECOG PS 0–1) were associated with better outcomes. Poor ECOG PS (≥2) was linked to shorter ToT and OS, regardless of age. Older patients were less likely to receive second-line therapy due to comorbidities and functional decline. These findings highlight the importance of performance status over chronological age in predicting benefit from immunotherapy and support pembrolizumab monotherapy as an effective first-line option for older patients with good functional status.

## 1. Introduction

Lung cancer remains a significant global health challenge, as it is the most frequently diagnosed cancer and the leading cause of cancer-related mortality worldwide [1]. Non-small-cell lung cancer (NSCLC) accounts for 80% to 85% of all lung cancer cases [2].

Data from the Surveillance, Epidemiology, and End Results (SEER) program (2017–2021) reveal that the highest incidence occurs in individuals aged 65 to 74 years, with 35.6% of new cases [3]. Additionally, 36.6% of cases occur in patients aged 75 or older according to data from the United States [3]. Moreover, the median age of death for patients with lung cancer is 73 years, underscoring the disproportionate burden of this disease on the elderly population.

Despite the high prevalence of NSCLC in patients aged 70 years or older, elderly individuals remain underrepresented in clinical trials, including those evaluating immunotherapy [4]. This discrepancy exists despite the promising advancements offered by immune checkpoint inhibitors (ICIs), which have revolutionized the treatment landscape for metastatic non-oncogene-addicted NSCLC. Pembrolizumab monotherapy, in particular, has demonstrated superior efficacy and tolerability compared to standard chemotherapy for the treatment of metastatic non-oncogene-addicted NSCLC patients with a programmed death-ligand 1 (PD-L1) tumor proportion score (TPS) ≥ 50% [5]. These findings established pembrolizumab as a cornerstone treatment in this setting [6].

The effectiveness of ICIs in the elderly is not uniform and may decline with advancing age, largely due to immunosenescence. This age-related decline and remodeling of immune functions have been hypothesized to limit the benefits of ICIs in patients aged 75 years and older, posing a challenge to optimizing treatment outcomes [7].

Although the age of 65 is commonly used as a cutoff in clinical trials to define “elderly,” geriatric assessments and cancer care guidelines frequently use 70 years as a threshold to identify and address the needs of older patients [4].

Given the aging global population and the high prevalence of NSCLC among older adults, there is a need to refine treatment strategies for this demographic.

This paper aims to evaluate the efficacy of pembrolizumab monotherapy in metastatic non-oncogene-addicted NSCLC patients with a PD-L1 TPS ≥ 50% aged 70 and older and compare it to patients younger than 70 based on real-world data collected from academic institutions in Central and Southeastern Europe. Furthermore, we aim to determine the clinicopathological characteristics that may be associated with favorable outcomes in this population.

## 2. Materials and Methods

### 2.1. Patients

This study included patients with histologically confirmed metastatic NSCLC with PD-L1 TPS ≥ 50% that started treatment with pembrolizumab monotherapy between December 2017 and October 2023. The cutoff date was 22 May 2024. The study was conducted at three academic European centers. There were 294 patients included from the Clinic for Pulmonology, University Clinical Centre of Serbia, 41 patients from the University Clinic Golnik, Slovenia, and 46 patients from several municipal hospitals in Vienna, Austria. Data was retrieved from hospital-based lung cancer registries.

All patients underwent routine PD-L1 testing performed on formalin-fixed, paraffin-embedded histology or cytology samples using PD-L1 monoclonal antibodies (22C3 clone by DAKO, Glostrup, Denmark, or SP263 clone by Ventana/Roche, Oro Valley, AZ) prior to initiating the first line of treatment. Patients in the Serbian cohort were tested for EGFR mutations by the Cobas^®^ EGFR Mutation Test v2 (Basel, Switzerland) and ALK rearrangements by immunohistochemistry prior to first-line treatment initiation. Patients in the Slovenian cohort were reflex-tested for EGFR mutations and ALK and ROS1 rearrangements in the same manner up until January 2022; after that, all the molecular tests were performed with the NGS Oncomine Precision Assay by ThermoFisher Scientific (Waltham, MA, USA). Patients in the Austrian cohort were also reflex-tested using NGS (Ion GeneStudio S5 ((Thermo Fisher Scientific) (Waltham, MA, USA))/Ion Torrent Oncomine Focus Assay) and simultaneous immunohistochemistry for ALK rearrangements.

Patients with detected driver oncogenes were excluded, so none of the included patients had a known driver oncogene. All patients were followed up with and assessed for the best response to therapy according to RECIST criteria according to local practice.

### 2.2. Ethics Approval

Data on patients with newly diagnosed lung cancer are prospectively collected within a clinical lung cancer registry that collects demographics and pathological and molecular characteristics, as well as treatment and survival data, of all patients with lung cancer diagnosed and treated at the center. All data were collected anonymously. The study was performed in accordance with the Declaration of Helsinki and approved by the Institutional Board Reviews and Ethics Committees of the institutions involved in the study (EK 20-061-VK; 602/3; 12 March 2024).

### 2.3. Statistical Analysis

Descriptive methods were used on the demographic characteristics of patients. Baseline information is presented as the number of patients and percentages. Median time on treatment (ToT) and overall survival (OS) were calculated as the time from the start of therapy to therapy discontinuation or the date of death. Patients still alive on the last day of follow-up were censored. Median ToT and OS were estimated by the Kaplan–Meier method and compared by the log-rank test. Univariable and multivariable Cox proportional hazard regression models were used to calculate hazard ratios (HRs) and confidence intervals (CIs). In the univariate analysis, covariates included age (<70 years vs. ≥70 years), sex, histology (non-squamous- vs. squamous-cell lung cancer), smoking status (current or former smoker vs. never-smoker), PD-L1 expression (TPS 50–79% vs. ≥80%), ECOG PS (0–1 vs. ≥2), and the presence of brain metastasis at baseline (yes vs. no). Multivariate analysis included variables with a significance level of *p* < 0.10 in the univariate analysis. The Chi-square test was used to determine the association between response to treatment and a patient’s age. Calculated *p*-values were two-sided. We used SPSS v26 for statistical analysis.

## 3. Results

There were 381 patients with metastatic NSCLC and a PD-L1 TPS ≥ 50% without detectable oncogene addiction that started treatment with pembrolizumab monotherapy up to 2023. Among them, the mean age was 66.2 (35–90) years, and 63.8% were male. There were 149 (39.1%) patients that were 70 years or older at the time of the treatment starting. Most of them were smokers or former smokers, 58.5% and 32.3%, respectively. Adenocarcinomas were detected in 66.4% and squamous-cell carcinomas in 23.1% of cases. There were 303 (79.5%) patients with good performance status, defined as Eastern Cooperative Oncology Group (ECOG) performance status (PS) 0–1 (Table 1).

Median time on treatment (mToT) and overall survival (mOS) for the whole cohort were 14.0 months (95% CI 10.5–17.5) and 22.63 (95% CI 16.7–28.6), respectively, at a median follow-up of 23.43 months. The best overall response to pembrolizumab was complete response, partial response, and stable disease in 3.1%, 33.3%, and 37%, respectively, adding up to an overall response rate (ORR) of 36.4% and disease control rate (DCR) of 73.4% (Table 1). Pembrolizumab was discontinued due to radiologically confirmed disease progression (215 patients; 56.4%), the development of high-grade immune-related adverse events (irAEs) (31 patients; 8.1%), or a decline in ECOG performance status, either from advanced NSCLC itself or from new or worsening comorbid conditions that precluded further immunotherapy (23 patients; 8%). An additional 24 patients (6.2%) completed the planned 2-year treatment duration, with their drug being discontinued at that time, but such instances were not included as an event in ToT analysis. There were no statistically significant differences in pembrolizumab discontinuation rates between patients aged <70 and those ≥70 years (*p* = 0.66), regardless of the reason for treatment cessation (Table 2).

**Figure 1:**
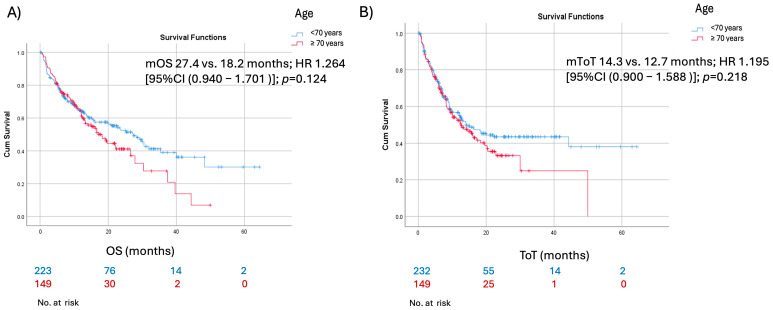
Kaplan–Meier survival curves of elderly patients and their younger counterparts for (**A**) overall survival and (**B**) time on treatment.

The comparison between patients younger than 70 years and those aged ≥70 years revealed no significant differences in clinical outcomes. The median time on treatment (mToT) was 14.3 months versus 12.7 months (*p* = 0.218; HR 1.195; 95% CI 0.900–1.588), and the median overall survival (mOS) was 27.4 months versus 18.2 months (*p* = 0.124; HR 1.264; 95% CI 0.940–1.701) (Figure 1). Similarly, no significant differences were observed in treatment response rates between the two groups (*p* = 0.55). (Table 3)

**Figure 2:**
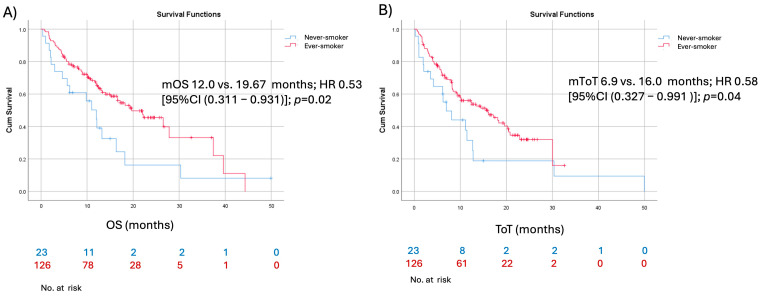
Kaplan–Meier survival curves comparing (**A**) overall survival and (**B**) time on treatment for elderly patients stratified by smoking status.

**Figure 3:**
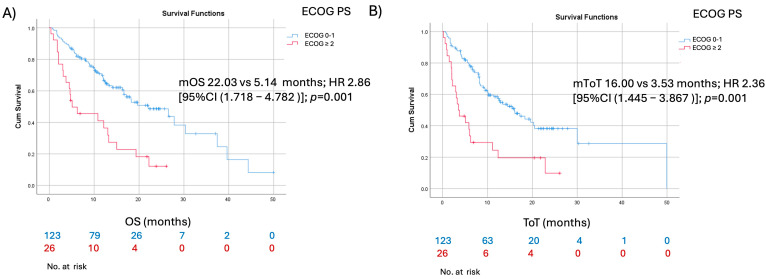
Kaplan–Meier survival curves comparing (**A**) overall survival and (**B**) time on treatment for elderly patients stratified by ECOG PS.

In the elderly patient group (aged ≥70 years), univariate analysis identified current or former smoking status and good performance status (ECOG PS 0–1) as factors associated with favorable time on treatment (ToT) and overall survival (OS). (Figure 2 and Figure 3) However, in multivariate analysis, only good performance status remained significantly associated with improved ToT and OS. Factors such as sex, histology, PD-L1 expression, and the presence of CNS metastases were not predictive of better ToT or OS (Table 4 and Table 5). Additionally, among 215 patients that experienced radiological PD, only 36 (16.7%) received systemic therapy in the second line. Patients were significantly more likely to receive treatment if they were younger than 70 years (26 patients vs. 10 patients; *p* = 0.04) (Table 6).

## 4. Discussion

Our results have shown that there was no significant difference in the efficacy of first-line pembrolizumab monotherapy in patients with metastatic NSCLC and a PD-L1 TPS ≥ 50% aged 70 and older when compared to their younger counterparts. Among the patients aged ≥70 years, a smoking history and ECOG PS 0–1 were associated with favorable outcomes in terms of ToT and OS. Multivariable analysis showed that ECOG PS 0–1 was both prognostic and predictive in this patient group.

Patients older and younger than 70 years had an equivalent ORR, ToT, and mOS. This is in line with the findings of other real-world-based data studies [8,9,10,11]. While some of these studies involved large cohorts, they lacked therapeutic consistency due to the inclusion of multiple immunotherapeutic agents and varying lines of treatment. To address this, we aimed to evaluate the impact of age on patients receiving pembrolizumab as a first-line therapy, similarly to the studies by Alessi et al. and Grosjean et al., but with a larger sample size [8,10].

In the older patient group (aged ≥70 years), univariate analysis identified current or former smoking status and good performance status (ECOG PS 0–1) as factors associated with favorable ToT and OS. The relationship between smoking status and treatment outcomes with ICIs remains a subject of debate. Ever-smokers have been found to exhibit higher tumor mutational burden (TMB), leading to increased neoantigen presentation and greater tumor immunogenicity, which can enhance ICI efficacy [12]. Additionally, smoking-related mutations may create a more inflamed tumor microenvironment with increased T-cell infiltration, further promoting the response to immunotherapy [13]. While some studies suggest that smoking may enhance the response to immunotherapy in patients with NSCLC, others present contradictory findings.

In a study by Popat et al., ever-smokers with advanced NSCLC and PD-L1 TPS ≥ 50% who initiated first-line pembrolizumab exhibited significantly prolonged OS compared to never-smokers [14]. Similarly, Cortellini et al. demonstrated that ever-smokers derived greater benefit in terms of OS and PFS compared to never-smokers [15]. Conversely, a recent meta-analysis of ICI-based registrational trials in advanced NSCLC found no association between smoking status and the efficacy of immunotherapy [16]. However, it is important to note that this meta-analysis included trials that enrolled patients with varying PD-L1 TPSs and evaluated different ICI-based regimens, including combination therapies. The discrepancies in findings mentioned above may be attributed to variations in study designs, patient populations, and treatment regimens. Factors such as differences in PD-L1 expression levels, tumor mutational burden, and the specific ICIs used could contribute to the inconsistent findings.

In our cohort, 20.5% of patients had ECOG PS ≥ 2. Previous reports suggest that patients with an ECOG of ≥2 comprise 30–48% of those with advanced NSCLC [17,18]. However, most pivotal trials of ICIs completely excluded this patient population [19]. A recent publication by Meyers et al. investigating the impact of ECOG PS on the treatment outcomes of patients with NSCLC undergoing ICI therapy reported that 29.2% of patients had ECOG PS ≥2 [20]. This study included patients that also received ICI therapy in the second line and further lines of treatment. The authors found that patients with ECOG PS > 2 were statistically more prevalent in the group of patients receiving ICI therapy in the second line and further lines of treatment, rather than as a first-line treatment. The relatively low proportion of patients with ECOG performance status (PS) ≥ 2 in our cohort (20.5%) is likely due to national prescribing and reimbursement policies. In Central and Southeastern Europe, first-line pembrolizumab monotherapy is generally approved only for patients with ECOG PS 0–1, consistent with regulatory indications and international guidelines. As a result, patients with poorer performance status were included only in exceptional circumstances. These access restrictions, common in many healthcare systems, contribute to the selection of patients with a better functional status who are more likely to tolerate and benefit from immunotherapy. Another more recent multicentric study that only included patients with advanced NSCLC and PD-L1 ≥ 50% that received pembrolizumab monotherapy in a first-line setting, as was the case with our cohort, reported a near-identical prevalence of ECOG PS ≥ 2 patients of 20.2% [21]. Similarly to our findings, Meyers et al. also reported no association between poor ECOG PS and older age [20].

A fairly limited number of studies have focused on patients with poor ECOG PS. Notably, registration trials for nivolumab [22,23], atezolizumab [24], and pembrolizumab [25,26,27] excluded individuals with an ECOG PS 2.

Data stemming from real-world studies has consistently shown that ECOG PS ≥ 2 has been found to be associated with worse treatment outcomes among patients with NSCLC undergoing ICI therapy [10,11,17,20,21,28,29,30,31,32,33]. This is in line with our findings where ECOG PS ≥ 2 was a negative prognostic factor and associated with shorter mToT and OS.

The PePS2 study demonstrated that checkpoint inhibitors could benefit patients with ECOG PS 2, showing outcomes comparable to registrational studies. The study included only patients with PS 2 (median age: 72 years, IQR 65–75) who received pembrolizumab monotherapy in either the first line or subsequent therapy lines. An important limitation was the absence of a control group with ECOG PS 0–1 [34].

Similarly, the IPSOS phase 3 trial provided important insights into treatment options for patients deemed ineligible for platinum-based chemotherapy due to poor ECOG PS ≥ 2, advanced age, or significant comorbidities [19]. In this trial, patients were randomized to receive either single-agent chemotherapy or atezolizumab as a first-line treatment regardless of the PD-L1 TPS. The results showed that atezolizumab improved median OS (10.3 vs. 9.2 months; *p* = 0.028). These findings reinforce the notion that while ECOG PS ≥ 2 remains a poor prognostic factor, mono-ICI therapy may still be a viable option for select patient populations with advanced NSCLC who cannot tolerate platinum-based regimens.

We found that older patients were less likely to receive second-line treatment following disease progression. After progression on first-line ICIs, second-line treatment with chemotherapy poses significant challenges. Advanced age is often accompanied by increased comorbidities, polypharmacy, diminished physiological reserve, and poor performance status at the time of disease progression, all of which can limit tolerance to further systemic treatment [35,36]. Due to the challenges posed by second-line therapy, clinicians sometimes opt to continue pembrolizumab beyond radiological progression if there is evidence of clinical benefit. This approach is both reasonable and proven to be efficacious, since radiological improvement is often preceded by a clinical enhancement of patient status. Additionally, immune-related or cumulative toxicities from first-line immunotherapy may preclude continuation with subsequent regimens. Cognitive or functional decline, social support limitations, and patient or caregiver preferences may also influence decisions to forego further therapy. To address this unmet need, individualized geriatric assessments, closer monitoring, and the early integration of supportive care are critical to preserving treatment eligibility. Furthermore, treatment sequencing strategies that maximize the benefit from first-line therapy—such as selecting patients with good ECOG PS and predictive biomarkers—are especially important in elderly populations, where opportunities for later-line interventions are often limited.

## 5. Conclusions

First-line pembrolizumab monotherapy is effective across all age groups and provides similar treatment efficacy in patients with metastatic NSCLC and PD-L1 TPS ≥ 50%. Patients older than 70 with a smoking history and good ECOG PS derive the most benefit from this treatment modality. The adaptation of this treatment strategy for elderly patients is especially important, since only a minority of them are capable of receiving second-line therapy.

## 6. Limitations

As a retrospective observational study, certain clinical information was either unavailable or inconsistently recorded in physician charting. Additionally, due to the retrospective nature of this study, we did not collect data on geriatric assessments and safety, which are particularly relevant when evaluating elderly populations. The absence of structured tools such as the G8 screening tool or comprehensive geriatric assessments limits our ability to assess frailty, functional status, and their influence on treatment decision-making and outcomes. Similarly, the lack of comprehensive toxicity data prevents us from evaluating not only treatment tolerability and potential trade-offs between efficacy and safety in this vulnerable group but also their capacity as potential prognostic and predictive markers of treatment response. Furthermore, for the purpose of analysis, age was treated as a dichotomous variable, whereas in reality, age is a continuous parameter, and this simplification might limit the nuance of our findings. Moreover, there are local differences in treatment approaches across the countries and centers included, making it more difficult to precisely determine some variables, such as the exact time of disease progression, etc.

The high proportion of censored cases may reflect several factors, including patients still on treatment at the time of data cutoff, limited follow-up duration, loss to follow-up, or incomplete documentation in medical records. In particular, a notable number of patients initiated treatment toward the end of the enrollment period, further contributing to shorter follow-up. These factors may have affected the accuracy of long-term estimates, including survival and time on treatment. While all patients were tested for the presence of EGFR and ALK driver mutations, only a subset underwent more comprehensive genomic testing. The Serbian and Slovenian cohorts initially relied on single-gene testing, whereas broader NGS panels were introduced later in the Slovenian cohort and were standard in the Austrian cohort throughout the study period. Heterogeneity in molecular profiling, along with incomplete data capture from NGS results, limited our ability to assess the prognostic or predictive relevance of other genomic alterations (e.g., STK11, KEAP1, KRAS). Consequently, molecular data beyond EGFR and ALK could not be systematically analyzed, which limited our ability to assess the prognostic and predictive capabilities of these findings.

## Figures and Tables

**Table 1:** Patient characteristics.

N = 381			N (%)
Mean age at treatment start (range) [years]			66.21 (35–90)
Aged ≥70 years			
Yes			149 (39.1)
No			232 (60.8)
	Patients ≥ 70 (N = 149)	Patients < 70 (N = 232)	*p* value
Sex			0.54
Male	84 (56.4%)	138 (59.5%)	
Female	65 (43.6%)	94 (40.5%)	
Smoking status			0.01
Current or former	123 (84.5%)	220 (94.8%)	
Never	26 (15.5%)	12 (5.2%)	
ECOG PS			0.35
0–1	123 (82.5%)	180 (77.6%)	
≥2	26 (17.5%)	52 (22.4%)	
Histology			0.88
Non-squamous	114 (76.5%)	179 (77.3%)	
Squamous	35 (23.5%)	53 (22.8%)	
PD-L1 status			0.37
50–79%	72 (48.3%)	123 (53%)	
80–100%	77 (51.7%)	109 (47%)	
CNS metastasis at baseline			0.07
Yes	23 (15.4%)	53 (22.8%)	
No	126 (84.6%)	179 (77.2)	
Radiotherapy			0.52
Yes	38 (25.5%)	66 (28.5%)	
No	111 (74.5)	166 (71.5%)	
PD as per RECIST			
Yes			215 (56.4)
Received systemic treatment in 2nd line of therapy			36 (16.7)
Did not receive systemic treatment in 2nd line of therapy			179 (83.3)
No			166 (43.6)
Best response to pembrolizumab (RECIST)			
PD			101 (26.6)
SD			141 (37.0)
PR			127 (33.3)
CR			12 (3.1)
Real-world DCR			279 (73.4)
Real-world ORR			138 (36.4)
Median ToT (95% confidence interval) [months]			14.0 (10.5–17.5)
Median overall survival (95% confidence interval) [months]			22.63 (16.7–28.6)

**Table 2:** Response rates of older and younger patients.

Discontinuation Reason	<70 Years (*n* = 167)	≥70 Years (*n* = 102)
Disease progression	129 (77.2%)	86 (84.3%)
High-grade irAEs	22 (13.2%)	9 (8.8%)
Decline in ECOG PS/comorbidities	16 (9.6%)	7 (6.9%)

**Table 3:** Response rates of older and younger patients.

	Age ≥70 Years (N = 149)-N (%)	Age <70 Years (N = 232)-N (%)
CR	6 (4.0%)	6 (2.6%)
PR	53 (35.6%)	74 (31.9%)
SD	51 (34.2%)	91 (39.2%)
PD	39 (26.2%)	61 (26.3%)

**Table 4:** Univariate regression analysis of elderly patients for ToT.

	Univariate Regression Analysis			Multivariate Regression Analysis		
	HR	95% CI	*p*	HR	95% CI	*p*
Radiotherapy (yes vs. no)	0.677	0.423–1.143	0.134			
Sex (male vs. female)	1.393	0.926–2.094	0.111			
Histology (non-squamous vs. squamous)	1.085	0.707–1.665	0.708			
Smoking status (current or former smoker vs. never-smoker)	0.581	0.327–0.991	0.048	0.645	0.379–1.098	0.108
PD-L1 (50–79 vs. 80%+)	0.979	0.648–1.480	0.920			
ECOG PS (0–1 vs. ≥2)	2.364	1.445–3.867	0.001	2.266	1.380–3.720	0.001
CNS mets (yes vs. no)	1.024	0.568–1.847	0.937			

**Table 5:** Univariate regression analysis of elderly patients for OS.

	Univariate Regression Analysis			Multivariate Regression Analysis		
	HR	95% CI	*p*	HR	95% CI	*p*
Radiotherapy (yes vs. no)	0.685	0.416–1.214	0.134			
Sex (male vs. female)	1.308	0.834–2.051	0.242			
Histology (non-squamous vs. squamous)	1.050	0.610–1.805	0.861			
Smoking status (current or former smoker vs. never-smoker)	0.538	0.311–0.931	0.027	1.104	0.8–1.524	0.546
PD-L1 (50–79 vs. 80%+)	0.992	0.644–1.530	0.973			
ECOG PS (0–1 vs. ≥2)	2.867	1.718–4.782	0.001	2.789	1.659–4.690	0.001
CNS mets (yes vs. no)	1.189	0.638–2.216	0.585			

**Table 6:** Patients receiving second-line systemic therapy following PD.

	Patients Receiving Second-Line Systemic Therapy Following PD as per RECIST (N = 215; 100%)	
	Yes (N = 36; 16.7%)	No (N = 179; 83.3%)
Age < 70 years	26 (12%)	83 (38.6%)
Age ≥ 70 years	10 (4.6%)	96 (44.4%)

## Data Availability

The dataset generated and analyzed during the current study is not publicly available due to privacy regulations and consent restrictions but are available from the corresponding author upon reasonable request. All data shared will be de-identified in accordance with ethical guidelines.
